# Critical pedagogical designs for SETS knowledge co-production: online peer- and problem-based learning by and for early career green infrastructure experts

**DOI:** 10.1186/s42854-023-00051-1

**Published:** 2023-04-03

**Authors:** Mathieu Feagan, Megan Fork, Geneva Gray, Maike Hamann, Jason K. Hawes, Elizabeth H. T. Hiroyasu, Brooke Wilkerson

**Affiliations:** 1 Department of Knowledge Integration, Faculty of Environment, University of Waterloo, 200 University Avenue West, Waterloo, ON N2L 3G1 Canada; 2 Department of Biology, West Chester University, 700 South High Street, West Chester, PA 19383 USA; 3 Department of Marine, Earth, and Atmospheric Sciences, North Carolina State University, 2800 Faucette Drive, 1125 Jordan Hall, Campus, Box 8208, Raleigh, NC 27695 USA; 4 Centre for Sustainability Transitions, Stellenbosch University, 19 Jonkershoek Rd, Stellenbosch, 7600 South Africa; 5 School for Environment and Sustainability, University of Michigan, Dana Building, 440 Church Street, Ann Arbor, MI 48109 USA; 6 The Nature Conservancy, California, 830 S Street, Sacramento, CA 95811 USA; 7 Department of Geography, System Dynamics Group, University of Bergen, P.O. Box 7800, 5020 Bergen, Norway; 8 Department of Geography, Centre for Climate and Energy Transformation, University of Bergen, P.O. Box 7800, 5020 Bergen, Norway

**Keywords:** Co-production, Critical pedagogy, Green infrastructure, Capacity building, Social-ecological-technological systems, Transformation, Online collaboration, Early career

## Abstract

Despite a growing understanding of the importance of knowledge co-production for just and sustainable urban transformations, early career green infrastructure experts typically lack opportunities to practice transdisciplinary knowledge co-production approaches within their normal training and professional development. However, using online collaboration technologies combined with peer- and problem-based learning can help address this gap by putting early career green infrastructure experts in charge of organizing their own knowledge co-production activities. Using the case study of an online symposia series focused on social-ecological-technological systems approaches to holistic green infrastructure implementation, we discuss how critical pedagogical designs help create favorable conditions for transdisciplinary knowledge co-production. Our work suggests that the early career position offers a unique standpoint from which to better understand the limitations of current institutional structures of expertise, with a view towards their transformation through collective action.

**Supplementary Information:**

The online version contains supplementary material available at 10.1186/s42854-023-00051-1.

## Science highlights

- ◦ Critical pedagogical designs help early career green infrastructure experts practice transdisciplinary knowledge co-production
- ◦ Online peer-led problem-based learning can support a social-ecological-technological systems approach to urban transformations
- ◦ Further research is warranted into critical and Indigenous pedagogies for shifting power and building institutional capacity for knowledge co-production

## Policy and practice recommendations

- ◦ Early career green infrastructure experts should explore how online collaboration tools help practice knowledge co-production across institutional contexts
- ◦ Institutions of higher education should support such exploration to accelerate capacity-building for transdisciplinary knowledge co-production

## Introduction

Within the context of sustainability transitions, knowledge co-production is increasingly recognized as necessary for addressing the complex intersections of climate change and urbanization (Frantzeskaki 2018). Defined as “iterative and collaborative processes involving diverse types of expertise, knowledge and actors to produce context-specific knowledge and pathways towards a sustainable future” (Norström et al. 2020), co-production is a core component in recent urban transformation case studies (Pereira et al. 2020; Buyana et al. 2021). However, implementing co-production in ways that transform dominant power relations remains an ongoing challenge (Avelino 2017; Pearsall et al. 2022), raising methodological and evaluative questions. Methodologically, who designs the co-production process, based on what ontological and epistemological starting points, who else is invited, when, and on what terms? Different possible responses to these questions lead to vastly different outcomes (Latulippe & Klenk 2020; Manuel-Navarrete et al. 2021; Chakraborty et al. 2022). Likewise in terms of evaluation, who is best positioned to measure the success of co-production? Here, too, there is a range of responses with various confirmations and doubts about co-production’s effects on catalyzing urban transformations (Perry & Atherton 2017; Palmer et al. 2020; Peris & Bosch 2020).

**Fig. 1:**
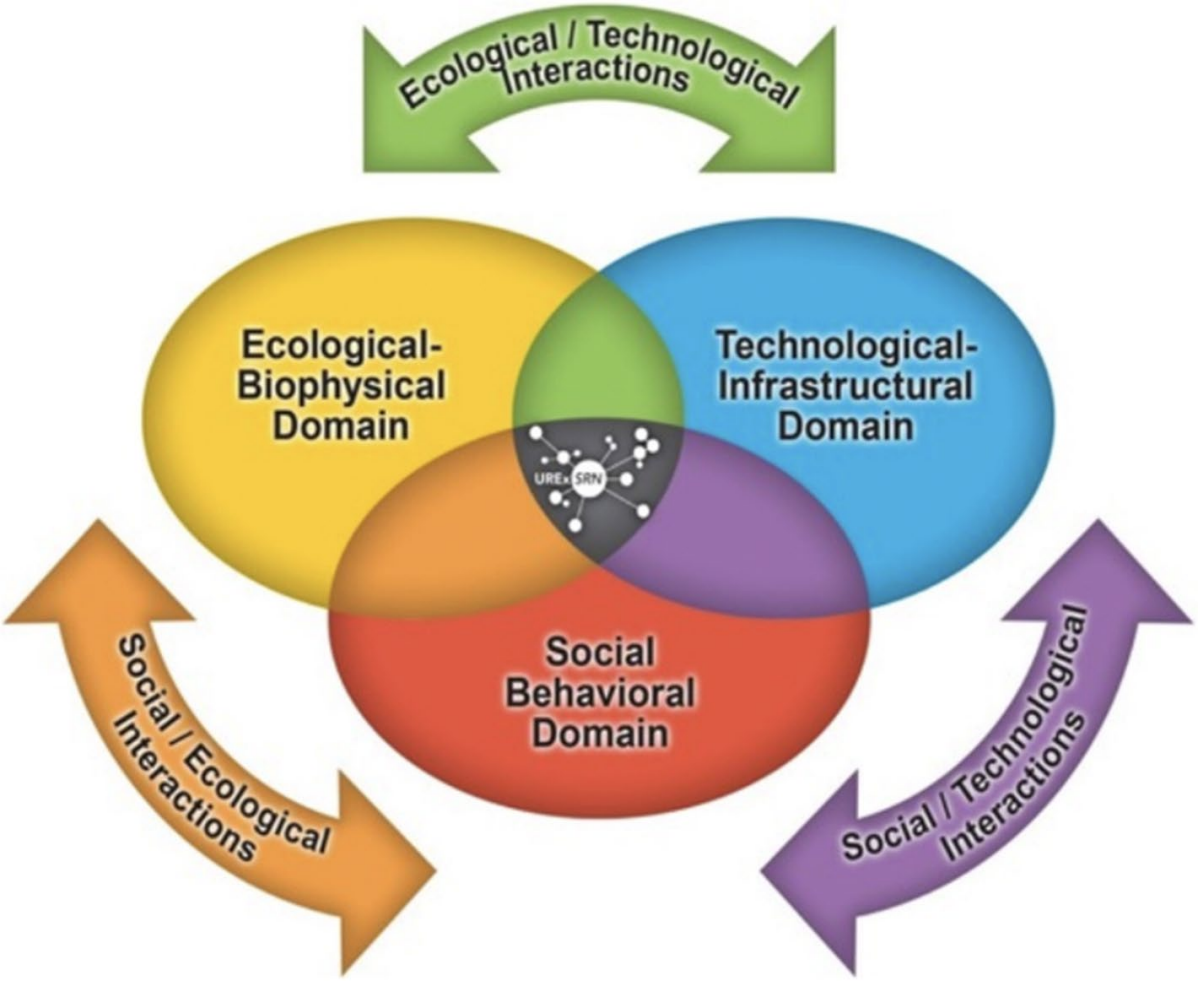
Interactions Across Social-Ecological-Technological Systems (SETS)

In this paper, we approach these methodological and evaluative questions – and their underlying challenge of defining and implementing a successful co-production process – from the standpoint of early career experts who, on two interrelated levels, are working across the social-ecological-technological systems (SETS) dimensions of green infrastructure (GI) (see Fig. 1). On a first level, these early career experts are working within a particular field of GI in which they have specialized knowledge, whether it be in green accounting (business and legal studies), safe-to-fail infrastructure (engineering), the history of urban greenspace (landscape architecture), or urban stream ecology, among other fields. On a second level, they also have knowledge about the institutional working conditions within which GI training and practice occur, whether through becoming an assistant professor in a particular academic department, interning at an engineering firm, volunteering with a non-profit organization, or holding a position in municipal government, for example. Yet, because of their early career status, they have not fully internalized the given institutionalized measures of success as their own—and this leaves open a window for questioning their chosen career ladder before deciding whether or how best to climb it.

We contend that, combined, these two levels can achieve a standpoint that offers a more complete understanding of GI across SETS interactions (Matsler et al. 2021; Markolf et al. 2018), while also providing new insights into the possibilities for implementing a knowledge co-production process that can effectively navigate and transform the dominant power relations that organize current knowledge-making practices across different areas of expertise. But the challenge is in how to do this ‘combining’. This is where the concept of co-production – by itself – does not automatically transform power. Rather, certain intentional pedagogical designs are required to guide the appropriate methodological and evaluative responses, without which transformative capacity cannot be built (Wolfram 2016).

Here, we draw on critical pedagogy^1^ to help guide and explain the implementation of co-production in ways that transform existing power relations and build transformative capacity. Critical pedagogy differs from pedagogy *tout court* by explicitly challenging the assumption that learning and teaching happen outside of power relations in society. The point for critical pedagogy is not simply to deliver content in more effective ways from the teacher to the learner, but rather to develop learners’ capacity to question and intervene in the current power relations that structure the relationship between their education (knowledge) and struggles for social emancipation (working conditions) (hooks 1994). Critical pedagogy is one form of intentional design that could help provide methodological and evaluative parameters for concretizing the co-production process to explicitly address dominant power relations. This article contributes to a clearer understanding of the connections between co-production and critical pedagogy, arguing that early career GI experts have a role to play in redesigning the conditions of their own training through practicing online peer-led problem-based learning.

We first explore links between critical pedagogy and knowledge co-production. Next, we present our action-research methodology and case study, sharing insights from our experiences as organizers and participants in an online peer- and problem-based symposia series designed by and for early career GI experts. We then underline the implications for SETS knowledge co-production, acknowledging certain limitations and future directions for our research.

### Co-production and critical pedagogy synergies

Whereas knowledge co-production arose largely from within centres of expertise and their struggle to manage the “wicked” nature of global environmental change through better science and policy frameworks (FitzGibbon & Mensah 2012; Fróes & Lasthein 2020; Schneider et al. 2021), critical pedagogy arose through the struggles of oppressed groups seeking social justice and emancipation (hooks 1994; Giroux 2022). Still, co-production and critical pedagogy both aim to address real-world complexities through creating safe-enough spaces (Chambers et al. 2021; Pereira et al. 2020) where different actors can (temporarily) step outside of their ‘normal’ institutionalized practices, to share insights and build a collective analysis of long-term institutional change and social transformation.

### Addressing real-world complexities

Both knowledge co-production and critical pedagogy address real-world problems through strategic interventions. For knowledge co-production, this involves recognizing that issues of sustainability are not contained within any one field of expertise, but rather require coordination across different institutionalized knowledge systems (Muñoz-Erickson et al. 2017). For critical pedagogy, this involves organizing training activities that present learners with some of the actual challenges of current institutional working conditions, to deepen their analysis (Dewey 1938). Both approaches recognize the value of specialized expertise but reject a simple transfer model, where knowledge is presumed to move from an authorized knower to an unknowing learner, in favour of co-learning and co-creation.

### Creating safe-enough spaces

The aims of knowledge co-production and critical pedagogy align in efforts to open safe-enough spaces (Chambers et al. 2021) where actors can experiment and learn outside the constraints of their institutional roles or ‘normal’ positions within dominant power relations. For knowledge co-production, this has meant inviting experts from different institutions and non-academic communities to step away from their daily work routines to engage in shadow networks and transition arenas where creative and innovative multi/inter/trans-disciplinary approaches are encouraged (Pereira et al. 2020; Frantzeskaki 2018). For critical pedagogy, this has meant developing an anti-oppression framework that challenges systems of exclusion (based on race, class, gender, etc.). in the production of expertise (hooks 1994; Giroux 2022). Both recognize that the status quo of normal workday activities presents obstacles for institutional transformation, and both seek to implement new group processes in which different perspectives and creative learning are valued.

### Long-term transformation

Knowledge co-production and critical pedagogy share a commitment to long-term institutional change. Whereas co-production tends to emphasize collaborative approaches, critical pedagogy tends to recognize the role of conflict in social change. Both approaches need to engage better with different ontological and epistemological starting points (Simpson 2017), and tensions remain around when iterative change amounts to transformation versus containment within the status quo (Schipper et al. 2019; Palmer et al. 2020). Institutional change requires more than a one-off event; it requires “scaffolding” and “intentional infrastructural support” for growing a “critical mass” of “change agents,” concepts that both knowledge co-production and critical pedagogy aim to advance (Palmer et al. 2020; Giroux 2022).

### Action-research methods

Action-research invites groups into an action-reflection cycle of inquiry to understand and intervene in the changing world around them (Adelman 1993; Tripp 2005). Action-research is conducted by the group about its own situation, blurring the line between the researchers and the researched (Feagan 2019). In our case, the authorship team is comprised of a subset of organizers and participants from the Get Ready, Get SETS: GI! symposia series. We formed as a group during the final symposium in November 2020 and subsequently embarked on a one-year conversation – a total of 9 online meetings from January to December 2021 – to better understand our experiences of the symposia series’ pedagogical designs (Table 1 in Supplementary materials captures quotes about the key features of the symposia series).

### Case study: online peer- and problem-based learning for SETS GI training

**Fig. 2:**
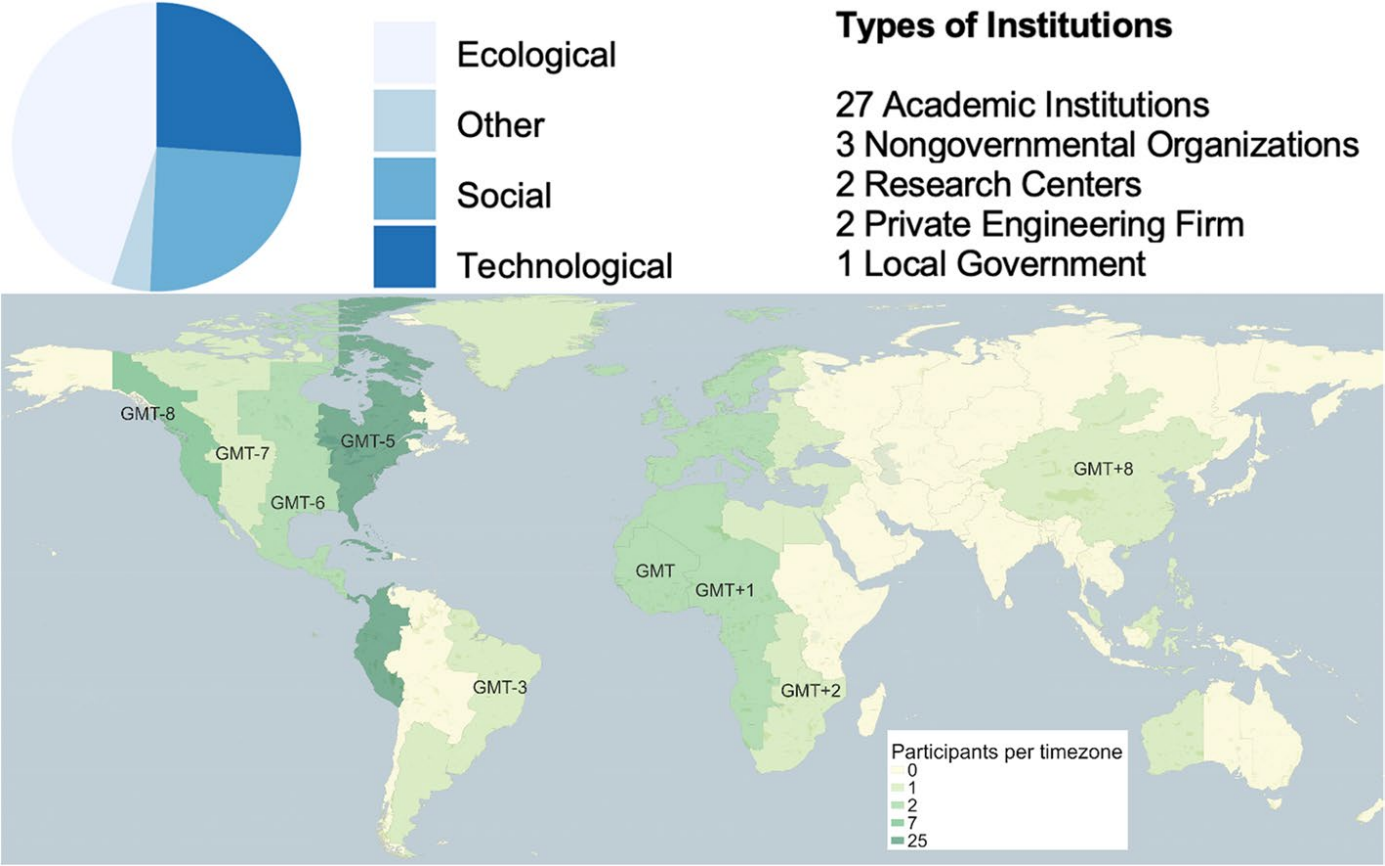
Time zone locations, institutional affiliations and SETS expertise of "Get Ready, Get SETS: GI! participants (The time-zone map is generated based on optional self-reported information from participants, but not all of the fifty-four participants chose to share this information)

The “Get Ready, Get SETS: GI!” symposia series was comprised of four online, interactive, half-day workshops delivered between July and November 2020, each building off of the previous one. The workshops were designed and run by and for early career experts, defined as anyone currently in or within five years of graduating from a graduate program, with an interest in one or more of the SETS dimensions of green infrastructure. The primary goals of the symposia series were (1) to form a network of support for the professional development of early career GI experts and (2) to create a set of guiding principles for using GI to address climate change in cities in holistic and transformative ways. Including the eight organizers, fifty-four early career participants took part from thirty-five institutions – primarily universities but also non-governmental organizations, engineering firms, government, and research institutes – located in six continents (see Fig. 2).

The symposia series met certain academic measures of success: it was a funded project focused on developing the next generation of leaders using a novel SETS approach, bringing together an international group of early career experts across different disciplinary fields. However, we argue that the symposia series’ critical pedagogical design point to three additional measures of success that enabled a suitable space for knowledge co-production: online collaboration, peer learning, and problem-based learning.

### Online collaboration

Typically, online collaboration is not the first element in critical pedagogical design, and pedagogy was not the only factor contributing to the decision to run the symposia series completely online.^2^ However, pedagogically, online collaboration offered a fundamentally different experience from a one-off in person event by creating opportunities for ongoing conversations across different institutional contexts and geographical regions. This was vital for the goal of building a network capable of learning across the SETS dimensions of GI that could endure beyond the symposia series itself. Zoom meetings, Google Docs, Mural boards, and Slack channels provided the virtual spaces for our activities. Although there is no replacing the value of embodied, in-person learning, being online offered a kind of virtual agora: an open space away from any one participant’s physical home turf, a place to interact because we are there together at the same time with shared interests in green infrastructure.

### Peer learning

But simply being online together was not enough—the second key element of the symposia’s critical pedagogical design was our peer learning model. In critical pedagogy, peer learning is not just a component used by the teacher to encourage student engagement, it is a strategy employed by learners to counter the individualizing effects of their training. Unlike normal graduate training where each individual student works with a supervisor within a prescribed disciplinary field while individually meeting certain established institutional requirements (e.g. coursework, comprehensive exams, and dissertation defense), peer learning teaches students how to work collectively across disciplinary boundaries, to define their own measures of success, and design their own learning activities for building transformative capacity. Along with valuing the group’s various forms of content expertise, peer learning places value on the group’s ability to shape the future conditions under which new versions of this expertise arise. Changing the conditions of GI training and practice can only be achieved through coordinated collective action, and early career peers are in a unique position to take on part of this action themselves.

The (temporary) absence of supervisors is worth underlining: we created new peer relations horizontally across the institutional silos and hierarchies that normally would divide us. This changed the way people interacted and the kinds of knowledge they shared. For example, participants talked about navigating pressures to fit GI into an engineering paradigm that left little room for the importance of community ownership over GI projects, and they talked about the disconnect between university research timelines and the time required for trust-building across different stakeholders. In short, the peer learning structure allowed participants to share expertise beyond their disciplinary training: they also brought expertise in how the institutional conditions under which GI is currently practiced undermined a more holistic approach.

### Problem-based learning

The third key element of our critical pedagogical design was problem-based learning (PBL).^3^ Instead of a lecture series by prominent leaders in the field, we organized our online peer interactions around building a collective analysis of a PBL scenario, drawing on our lived experiences working in various cities, universities, and other institutions. PBL offered a common focal point, as well as a set of steps, to direct our peer knowledge to addressing a complex and dynamic scenario (available in Table 2 of the Supplementary materials). After reading the scenario aloud in small group breakouts, teams analyzed the facts and assumptions and proposed a way forward. In a large group report back, we heard what each group was thinking, which provided further opportunities to discuss assumptions and compare the advantages and disadvantages of different possible approaches.

We also brought in outside experts to support our peer-learning and PBL approach, including community groups who shared place-based knowledge of holistic GI, faculty and other experts who participated in a happy hour exploring career opportunities, and a visualization expert who graphically depicted the major elements of each symposium. Each of these experts was invited to help us better engage with our focus on developing capacity for holistic GI, not as a replacement for the knowledge we were generating ourselves.

**Fig. 3:**
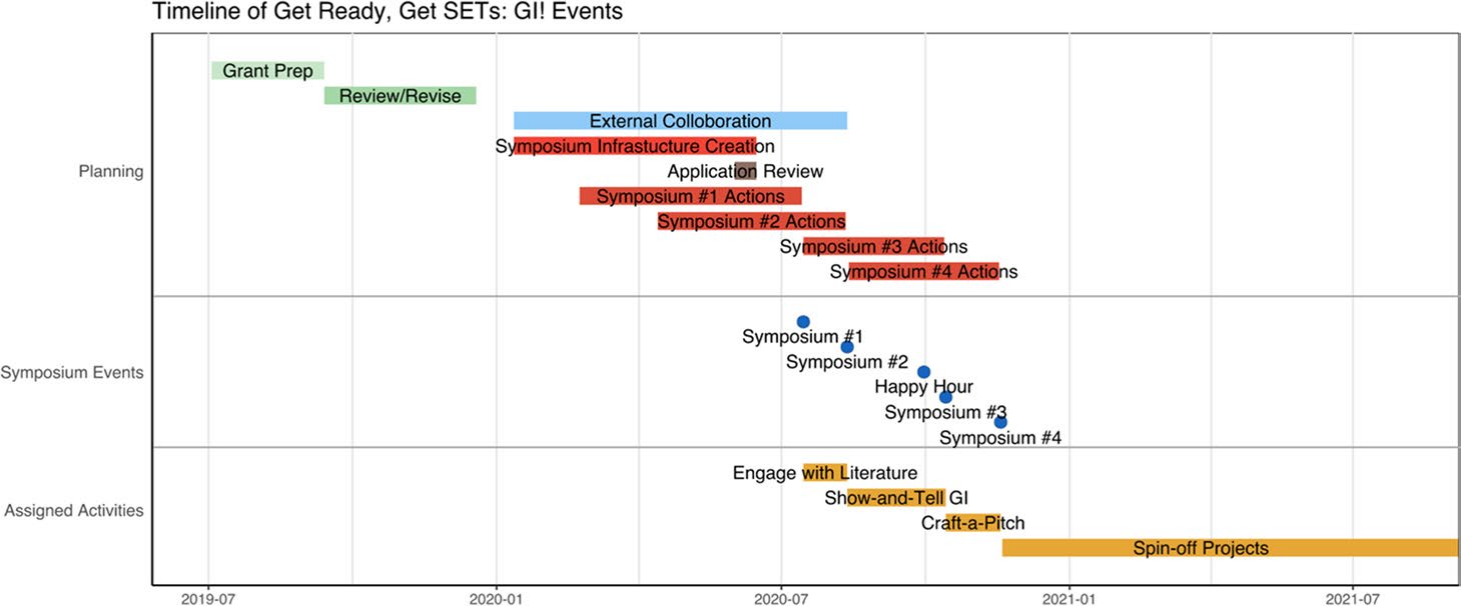
Timeline of activities surrounding the “Get Ready, Get SETS: GI!” symposia series. Proposal development (green); collaborations with external colleagues and institutions (blue); selected applications from participants (brown); format design of symposia activities (red). When participants reached the final symposium in November 2020 (blue circles), ongoing engagement activities (orange) were initiated

**Fig. 4:**
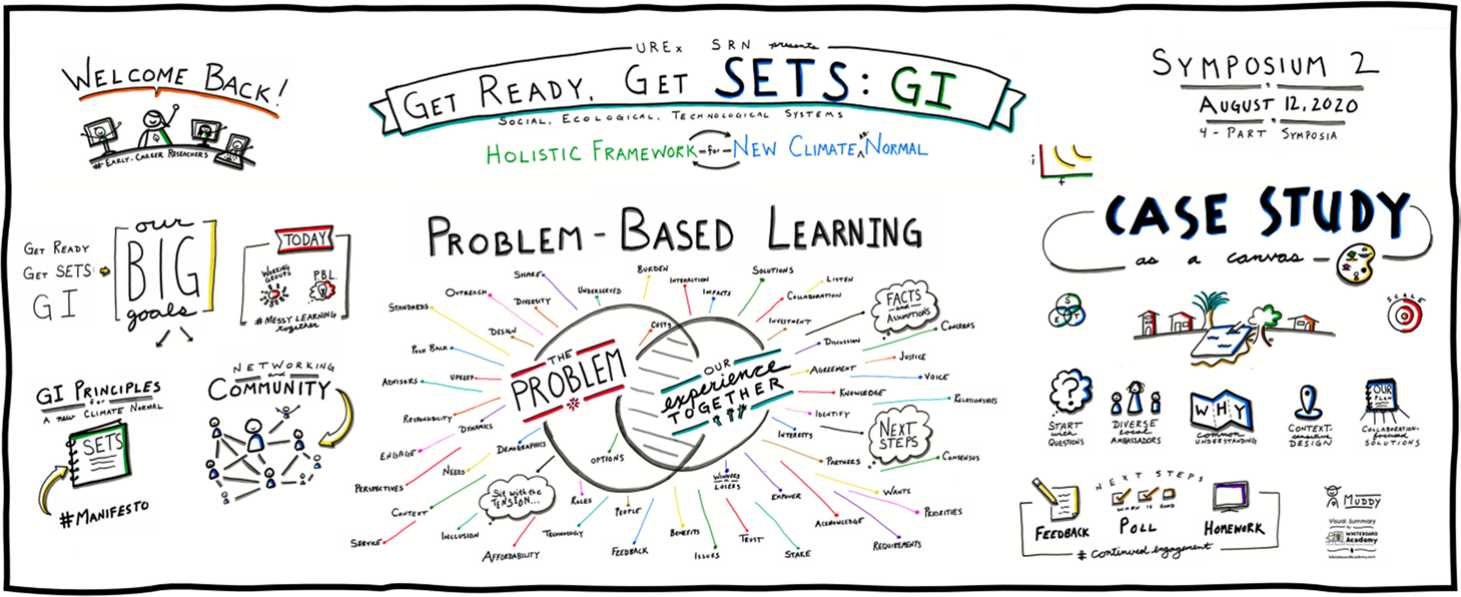
A visual summary of Symposium 2, the launch of the problem-based learning scenario and case study

In sum, the basic arc of the symposia series included (see Fig. 3): in symposium 1, we met, shared definitions of key terms such as GI and SETS, and discussed the goals of our collaboration; in symposium 2, we launched the first part of the problem-based learning scenario and explored the Good Neighbor Stormwater Park case study (see Fig. 4); between symposia 2 and 3 we held the happy hour networking event; in symposium 3, we worked on the second part of the PBL scenario to begin identifying the principles guiding what holistic GI implementation entailed; finally, in symposium 4, we presented a first draft of our guiding principles and opened the floor for new project pitches to continue collaborating. At the end of each symposium, a Google form was provided for individual feedback and reflection on key learning, with room to make suggestions for the next symposium. The organizing team read this feedback and made changes to the symposia design accordingly. By the end of the symposia series, participants had built capacity for lasting engagement in transdisciplinary knowledge co-production, as groups launched new projects which continue now, two years later.

### Implications for SETS knowledge co-production

Based on our case study, we now show how critical pedagogical designs are relevant for supporting a SETS co-production process that considers power relations. Although we have discussed online peer- and problem-based learning sequentially, these are deeply interconnected and mutually supporting elements within the Get SETS’ critical pedagogical design (see Table 1).

**Table 1:** Critical pedagogical elements as they appear in the Get SETS symposia design

**Elements of critical pedagogy** (Freire 2000 & 2005; hooks 1994; Giroux 2022)	**Where these factor into the Get SETS symposia design**
Critical pedagogy emphasises that learning goes beyond the individualized approaches of the banking model of education, towards enabling a group to build its capacity to collectively intervene in the conditions of its own practice	**Online collaboration** enabled multiple encounters to increase opportunities for building trust and planning further group collaborations. A one-off event cannot compare to the transformative potential of ongoing working relationships
Critical pedagogy takes into account the existing power relations organizing the structures within which people encounter the world, suggesting that emancipatory education requires learners to develop an awareness of both content expertise and the conditions that allow such expertise to come into existence	**Peer learning** happens within a given stratum of wider institutional power relations. By taking more senior experts out of the room and putting early career experts in a position to learn from each other, peer learning shifts dominant power relations and opens a space for peers to reflect on their capacity to design and implement their own learning activities
Critical pedagogy takes a problem-posing approach, in which the group must confront a real-world problem relevant to their experience while simultaneously reflecting on its own framings of the problem, challenging dominant assumptions and developing critical consciousness	**Problem-based learning** ‘forces’ participants to compare different possible framings of the problem, collectivize their analysis, and plan steps forward. Participants are encouraged to develop a group process that also reflects the kinds of recommendations they are advancing in relation to the PBL scenario

### Online collaboration for long-term transformation

First, our case study suggests that online collaboration technologies help open a space outside of any one person’s institutional home for ongoing, cross-institutional dialogue in ways that are not possible in-person. The Get SETS symposia series produced not only a new set of principles for holistic GI implementation but also a new network of people ready to develop further projects together. With respect to long-term institutional transformation, such online networks can play a supportive role by providing a taste of a different set of learning conditions from those normally experienced in graduate and professional practice (Fork et al. 2021).

### Peer learning as safe-enough space

Secondly, SETS knowledge co-production requires a context-specific pedagogical design for interrupting dominant power relations to allow new knowledge-sharing practices to emerge. Holistic approaches to GI are currently limited by the very structures of GI training, which teach individual disciplinary experts to stay within and reinforce their separate disciplinary practices. Peer learning helps challenge the artificiality of these disciplinary boundaries, combining content expertise with knowledge about the need to self-organize networks that support a new capacity for transdisciplinary knowledge co-production. People in early career positions need safe-enough spaces to contemplate ways of forming these networks to build capacity for SETS co-production. Nothing prevents participants from voicing the ideas of their bosses and supervisors, but the peer group must now take responsibility for standing with or challenging these ideas. In this way, peer learning allows a different kind of horizontal transdisciplinary knowledge co-production, where alternatives to current siloed path dependencies can be evaluated by those who must either accept dominant knowledge-making practices or work to change them.

### Problem-based learning for addressing real-world complexities

Thirdly, in response to sustainability science’s calls for “agile scientists” and “change agents” capable of “epistemic pluralism” for addressing real-world complexities (Miller et al. 2008; Kueffer et al. 2012; Haider et al. 2018; Yeung et al. 2021), problem-based learning offers a methodology for putting the theory of co-production into practice, without relying on any ready-made heuristic, conceptual framework, or set of values already deemed ‘correct’ by existing authorities. Early career experts must learn how to confront dynamic real-world complexities through their own action and group reflection, and PBL encourages this action-reflection on two levels. At the level of the PBL scenario, participants spend time assessing the problem and unpacking the assumptions they bring to understanding its frame. Simultaneously, participants are confronted with the other reality outside of the PBL scenario, that is their own group process: how are they exercising agency in ways that embody or are consistent with the kinds of recommendations they are making to the protagonist in the PBL scenario? By assessing this correspondence between the two levels, PBL invites learners to confront the changes that must happen both *in themselves* and in their *working conditions* somewhat simultaneously.

### Limitations and further research

We acknowledge certain limitations to the present study, which can help identify directions for further research. First, in this paper we define capacity as our group’s ability to organize co-production learning activities that intentionally apply critical pedagogical designs in structuring how transdisciplinary knowledge co-production happens for early career GI experts. This definition aligns with critical pedagogy’s emphasis on a learn-by-doing approach to building a group’s capacity to intervene in the conditions of its own learning through ongoing action and reflection (Freire 2000; Dewey 1938). Transformative capacity is about a group’s ability to develop its own goals for a strategic orientation to working across scales to intentionally shape the conditions of future development, in part through education and training (Wolfram 2016). However these are not the only definitions of capacity, nor are they the only pedagogical designs available. For example, there are important discussions about “red pedagogy” and “land as pedagogy” that are relevant to exploring the similarities and differences between critical and Indigenous designs for knowledge co-production (e.g. see Denzin et al. 2008).

Second, although we build on criticisms of current disciplinary norms in graduate training and professional practice, institutions themselves need not be considered inherently oppressive to transdisciplinary knowledge co-production, as institutional supports are part of what enables different forms of expertise to work together. This implies a need for further research into successful examples of institutional arrangements that are currently supporting transdisciplinary approaches and how these might be replicated or scaled up (Kessel & Rosenfield 2008; Pearsall et al. 2022). For example, various pedagogical designs center the knowledge of affected communities (Corburn et al. 2021), explore different possible theories of change (Armitage et al. 2019), and re-organize graduate education to intentionally bring together young scholars across fields (Welch-Devine et al. 2014). We propose that online peer-led PBL could play a complementary role in advancing these efforts.

Finally, given that online communities can form and disappear nearly instantly, further research is needed in understanding the role of online collaboration in long-term transformative efforts (see Simpson 2017, chapter 12). In our case, further research with all fifty-four participants in the Get SETS symposia series would help better understand just what capacity has been built now two years since the symposia series ended.

## Conclusion

In the context of sustainability transitions, more explicit engagement with critical pedagogical designs could help shift the power relations of dominant knowledge-making practices, opening new possibilities for transdisciplinary knowledge co-production to contribute to urban transformations. More specifically, we have argued that online peer- and problem-based learning can open a critical space for early career experts to organize their own co-production activities and build skills as members of transdisciplinary teams across the social-ecological-technological systems (SETS) dimensions of green infrastructure implementation. Such a space is generally missing from the disciplinary and siloed approaches to university-based training and professional practice but early career experts have a role to play in building their own capacity for SETS knowledge co-production, while collectively reframing their responsibilities in generating new conditions for the future of holistic GI implementation.

## Supplementary Information

**Additional file 1.**

## Data Availability

The Supplementary Materials file contains additional data supporting the conclusions drawn in this article.

## Abbreviations

SETS: Social-ecological-technological systems
GI: Green infrastructure
PBL: Problem-based learning

## Footnotes

1 Critical pedagogy is an educational philosophy associated with the work of Brazilian educator Paulo Freire (2000; 2005) who, building on traditions of pragmatism (Dewey) and critical theory (from Karl Marx through to the Frankfurt School), developed an approach to literacy training through supporting the learners’ own social emancipatory struggle. In contrast to the “banking model” of education, in which the instructor deposits pre-formed content-knowledge that students later cash-in on exams in exchange for a grade/degree, Freire used a problem-posing approach in which students had to develop and apply their own knowledge, or “critical consciousness,” through action, reflection, and dialogue.

2 Staying within our $20 K budget and keeping a low carbon footprint were also factors pushing towards online collaboration—a decision reached prior to the COVID-19 pandemic.

3 PBL was formalized at McMaster University’s medical school as a way of better preparing medical students for working with the actual complexities and real-world dynamics of achieving strong health outcomes for patients, see Barrows (1996).
