# Supplementary material for: Critical pedagogical designs for SETS knowledge co-production: online peer- and problem-based learning by and for early career green infrastructure experts
Source: Urban Transform. 2023 Apr 3;5(1):6. doi: 10.1186/s42854-023-00051-1 (PMC10068209; doi:10.1186/s42854-023-00051-1)
Supplement: Supplementary file 1 — Additional file 1. [file 42854_2023_51_MOESM1_ESM.docx]

Supplementary Materials

**Table 1**: Features of the Get SETS GI! Symposia Series

| **Feature** | **Why it was effective** |
| --- | --- |
| Individual short reflection questionnaires on Google Forms | “As I recall this happened multiple times during the workshops, as well as after, where the participants were asked to take some time and reflect on what they’d learned and note it down in an online (and anonymous, if wanted) survey/ questionnaire. I thought this was a great way to get people to sit with their thoughts for a bit, and maybe distill some of the highlights and learnings from their point of view. It strikes me as a great way to get feedback and insights from a large group of people where not everyone will get an opportunity to voice their thoughts. It also made me as a participant feel like I was contributing to the process, even if I hadn’t said anything in plenary. I think the fact that it was “alone time” and structured by the questionnaire helped a lot. Other processes I’ve been a part of (and it was done in these workshops too) let you make notes or add thoughts on virtual whiteboards, but this felt more encouraging of reflexivity and personal insights (instead of trying to capture highlights from a group conversation).” |
| Structured Mural boards | “This was really great and allowed multiple ideas to be put out there really quickly. I was grateful how well organized the boards were and have implemented them in my own workshop facilitation” |
| Path self-determination | “Allowing folks to choose their breakout groups on the fly - important to continued engagement – I chose to be here, so I might as well be HERE, so to speak” |
| Cameras on or off | “One of the days of the symposium was two days before I closed on a house, and … things were not going smoothly (do they ever with house-buying?). This was one of the highlights of a very stressful week, but I could only participate because y’all accepted mental engagement and not visual or active engagement. Lots of other examples of why this is important, just thought I’d share mine.” |
| Free online platforms with no download required | “I participated on my old laptop most of the time. I’ve tried that in some workshops where they then said “ok, we’re going to test-drive this piece of software that is super important for the field.” And that was the end of my participation. Another way of looking at this - just no downloaded softwares in general seems like a safer option.” |
| Shared Google Doc as portal with all links and materials for each symposium | “I was really pleased with the organization of the google docs - it was clear where to click and made it easy to find things and re-find them easily as necessary. I found the curation, presentation, and sharing of the materials really streamlined and helpful. Indeed, I just copied this approach for a Zoom-based teaching demonstration I did as part of a job interview.” |
| Zoom chat | “I found the active zoom chat discussion during the sessions very engaging to follow along with how other people were reacting to the discussion. It made the experience more social, and people with specific competence to the topic posted relevant resources.” |
| Mix of session formats | “A mix of short, medium, and long sessions, in groups and in plenary etc., kept things interesting.” |
| Large organizing team | “It felt like the large organizing team had things under control, with lots of different roles/people that were in charge of different aspects of the event. It conveys a sense of confidence and as a participant I didn’t have that cringe-y feeling when there isn’t sufficient support to run a meeting efficiently and effectively” |
| Extended timeline online | “We certainly could not have gathered this group 4 times in person. Most similar NSF workshops are 2-3 days in person, long hours, many folks. Is this longer, extended timeline more productive? Did we enable network creation by forcing interaction over a period of months?? I’m not sure. Just another observation.  A possible critique: Does this reinforce skew to the Global North?” |

**Table 2**: Problem-based learning scenario parts 1&2

**Responding to the new the climate normal: PART I**

| *Instructions:* Have one person in your group share this screen. That person can start reading aloud the scenario below, with a new group member jumping in to play each of the new characters introduced. Once you’ve finished reading the scenario aloud, stop sharing this screen and respond collectively to the prompts at the bottom of the page. |
| --- |

**Narrator**: *It is 7:30pm. Two dozen people are scattered throughout the City Hall meeting room to hear proposals in response to the FOREVER FUTURES funding program, which aims to support green infrastructure projects in low-lying, flood-prone areas, to stimulate new development and accelerate the transition to resilience and sustainability. Several properties in the Lower North Shore neighborhood have been deemed “severe repetitive losses'' due to high-tide flooding. A representative from TipTop Condos has just presented a proposal entitled “Reaching New Heights,” which would raise the entire block by 4 feet and replace the existing properties with a 15 story condominium complex including diverse shopping and amenities, a private gym and pool facility, and 105 deluxe apartments with ocean views. Equipped with a system of sensors and pumps for removing flood water, the complex would provide residents access to a state-of-the-art smart monitoring network collecting real-time data about flood conditions with 24/7 safety alert notices. Now it is time to hear the next proposal to be presented by Holistic Design Solutions -- Chandra, has a Master’s degree in Landscape Architecture and was recently hired to assist with this proposal. She steps up to the microphone…*

**Chandra:** Dear residents of the Lower North Shore and members of The City Planning and Economic Development Board, we face a dilemma today that will play itself out in countless other neighborhoods across the state, as the water table continues to rise and the new climate normal imposes more intense and frequent extreme weather events on our lives. I get it -- you don’t want your properties to flood anymore. So, we can continue development as usual or we can begin to think long term about the needs of residents -- renters and landlords alike -- for sustainable and affordable options in order to continue dwelling in this beautiful peninsula we call home. With this in mind, *Holistic Design Firm* proposes a multi-functional green infrastructure project to minimize the effects of chronic flooding through the creation of a retention basin designed to manage the 25 year storm, complete with a meadow plant palette that leverages native species and attracts pollinators and signage explaining the functionality and benefits of the project. We want this community to live better, stay in place, and build local capacity for long-term flood resilience!

*[Chandra finishes her pitch confidently; the meeting chair thanks both presenters for their exceptional proposals and opens the floor for questions.]*

**Bruce (City employee and member of the mayor’s sustainability office):** Chandra, our local stormwater management requirements only require us designing for the 2 year storm for runoff volume- that would be a lot cheaper, and save space on the site for other uses. Why has your firm chosen 25 years?

**Chandra**: Designing for the 25-years storm does have higher upfront costs; however, it will save the city more money in the long term -- these repetitive loss properties demonstrate a need to be proactive and start taking the future of climate change seriously.

**Breann (Community member):** What we need is stronger rent control--neither proposal is talking about that and we’re sick of being pushed out to make room for richer families.

**Chandra:** Cost of living is a huge concern. *Holistic Design Solutions* is committed to working with residents to keep housing affordable--and green infrastructure can be part of the plan. This community is historically underserved and it’s one of the hottest and most flood prone areas of the city--more vegetation will help with that, while offering other co-benefits as well.

**Alice (Community member):** I don’t know about all these weeds you’ve got planned, I’ve got allergies--and who’s going to take care of this anyways? I’d prefer a clean mowed lawn.

*[Before Chandra can respond about the low maintenance costs and other benefits of native plants, the City Hall meeting doors burst open to a flow of protesters chanting “No more climate gentrification, no more climate gentrification!”]*

_______________________________

PROMPTS:

Now that you’ve finished reading the scenario aloud, stop sharing this screen, assign a note taker (you can take notes directly in this document) and respond collectively to the prompts below (you will have approximately 25 minutes):

- Group members’ names:
- Note taker’s name:
- Questions:
  - What do you agree are the main facts of the scenario?
    - Key Facts:
  - Would any of your facts be better described as assumptions?
    - List the basic assumptions you’re making:
  - What should Chandra do next?
    - Propose immediate next steps in the moment as well as a strategy for the coming weeks:
      - Immediate next steps:
      - Longer term strategy:

**Responding to the new the climate normal: PART II**

| *Instructions:* Have one person in your group share this screen and read Part II of the problem aloud, then respond as a group to the prompts below. |
| --- |

**Narrator**: *The protesters successfully called for a new planning process, starting with a series of community meetings to begin next week at a local church in the Lower North Shore neighborhood to better hear and understand the community’s needs and concerns. Chandra is now back at her office, her boss comes over to her desk…*

**BOSS***:* Chandra, some good news amidst the chaos -- the Mayor just called and here’s the deal: she wants *Holistic Design Solutions* to develop a new integrative plan that responds to the community’s concerns about flooding and affordable housing -- there’s a lot of pressure to make sure that this FOREVER FUTURES program is seen as a success, meaning a way for the city to practice sustainable growth in the face of climate change and flooding.

**Chandra**: Okay….

**BOSS**: The community meetings begin next week and the Mayor wants to hear our plan before then -- can you start coming up with a game plan so we’re ready? We need clear design priorities and a strategy for working with the community -- remember if this is seen as successful in the mayor’s eyes, it could lead to more opportunities. I have a phone call now, but can you meet me in my office in 30 minutes with some ideas to get us started in the right direction?

**Chandra**: Yes and --

**BOSS**: Okay great, I really have to run now, talk to you soon.

______________________________________________________________________

PROMPTS

- Write your group member names here:
  - …
  - ....
  - ...
- You now have just under 30 minutes to prepare: what will you say to the BOSS? If needed, you can review Part I of the problem from Symposium 2 and you can see a summary of notes. Be prepared to give your presentation and hear from two other groups as part of a report back. You might want to consider:

1. What is the heart of the problem?
2. What intervention should Chandra make?
3. Can you identify any guiding principles for holistic GI implementation?

- Designate a report back person:
